# Reprogramming of Round Spermatids by the Germinal Vesicle Cytoplasm in Mice

**DOI:** 10.1371/journal.pone.0078437

**Published:** 2013-10-22

**Authors:** Peng-Cheng Kong, Yan Zhu, Mei-Shan Wang, He-Ping Li, Xue-Jin Chen, Man-Xi Jiang

**Affiliations:** 1 Department of Laboratory Animal Sciences, Shanghai Jiao Tong University School of Medicine, Shanghai, China; 2 Key Laboratory of Contraceptive Drugs and Devices of National Population and Family Planning Committee, Shanghai Institute of Planned Parenthood Research, Shanghai, China; 3 College of Wildlife Resource, Northeast Forestry University, Harbin, China; University of Connecticut, United States of America

## Abstract

The birthrate following round spermatid injection (ROSI) remains low in current and evidence suggests that factors in the germinal vesicle (GV) cytoplasm and certain substances in the GV such as the nucleolus might be responsible for genomic reprogramming and embryonic development. However, little is known whether the reprogramming factors in GV oocyte cytoplasm and/or nucleolus in GV are beneficial to the reprogramming of round spermatids and development of ROSI embryos. Here, round spermatids were treated with GV cytolysates and injected this round spermatid alone or co-injected with GV oocyte nucleolus into mature metaphase II oocytes. Subsequent embryonic development was assessed morphologically and by *Oct4* expression in blastocysts. There was no significant difference between experimental groups at the zygote to four-cell development stages. Blastocysts derived from oocytes which were injected with cytolysate treated-round spermatid alone or co-injected with nucleoli injection yielded 63.6% and 70.3% high quality embryos, respectively; comparable to blastocysts derived by intracytoplasmic sperm injection (ICSI), but higher than these oocytes which were co-injected with lysis buffer-treated round spermatids and nucleoli or injected with the lysis buffer-treated round spermatids alone. Furthermore, the proportion of live offspring resulting from oocytes which were co-injected with cytolysate treated-round spermatids and nucleoli or injected with cytolysate treated-round spermatids alone was higher than those were injected with lysis buffer treated-round spermaids, but comparable with the ICSI group. Our results demonstrate that factors from the GV cytoplasm improve round spermatid reprogramming, and while injection of the extra nucleolus does not obviously improve reprogramming its potential contribution, although which cannot be definitively excluded. Thus, some reprogramming factors are evidently present in GV oocyte cytoplasm and could significantly facilitate ROSI technology, while the nucleolus in GV seems also having a potential to improve reprogramming of round spermatids.

## Introduction

Live offspring can be produced following round spermatid injection (ROSI) into the oocytes of mice, rats, hamsters, mastomys, rabbits and humans [1-7]. While this assisted reproduction technique allows the full-term development of embryos derived by ROSI, the birthrates are lower than those following intracytoplasmic sperm injection (ICSI; [3-5,8]. It has been shown that mouse embryos generated by ROSI displayed normal expression of six imprinted genes (three maternally expressed, three paternally expressed), which indicates that these spermatids have normal imprinting patterns [9]. However, several recent studies have reported abnormal gene expression patterns [10] and epigenetic modifications in ROSI-derived embryos [11]. The link between these changes and the low birthrate associated with ROSI remains unclear.

In mammalian cloning, enucleated metaphase II (MII) oocytes can be used to generate cloned offspring after injection with somatic or embryonic stem cell nuclei; however, only oocytes enucleated at MII or soon after pre-activation have been shown to fully reprogram transferred nuclei[12,13]. The germinal vesicle (GV) oocyte is thought to be a superior nuclear recipient for cloning, and mouse immature oocytes, but not MII oocytes can reprogram the immature nucleus [14,15]. It has also been proposed that passing the donor nucleus through the cytoplasm of the oocyte at these earlier stages of oogenesis might further improve reprogramming [15]. On the contrary, some studies have suggested that GV oocytes were unsuitable as recipients for nuclear transfer because the removal of the GV before nuclear envelope breakdown and meiotic metaphase arrest lead to abnormal cell division, with some crucial nuclear factors being removed from the GV oocytes during enucleation, so that the remaining cytoplasm could no longer support cloned embryo development [16-18]. These studies were unable to definitively exclude the presence of reprogramming factors in the GV ooplasm, and other reports[19,20] have demonstrated that the cytoplasmic lysates of GV oocytes can promote somatic cell reprogramming and cloned embryonic development, suggesting that factors in the cytoplasm of GV oocytes are required for genomic reprogramming. 

In addition to factors in the GV cytoplasm, it is also thought that the nucleolus of the oocyte is important for embryonic development. The nucleolus of spermatozoa are eliminated during spermiogenesis [21-23], so the oocyte nucleolar material is essential for the reassembly of newly formed nucleoli in both female and male pronuclei. Ogushi et al. reported that the nucleolus of the GV stage nucleus is essential for further embryonic development, comprising one of the factors essential for the reprogramming of somatic cell nuclei [24].

To date, the impact of the proposed reprogramming factors in the cytoplasm and nucleolus of the GV on the reprogramming of spermatids in MII oocytes and the development of ROSI embryos has not been studied. With this in mind, we studied the embryonic development of mature MII oocytes which were co-injected with GV cytolysate treated-round spermatids and GV oocyte nucleoli or injected with cytolysate treated-round spermatids alone. Our data suggests that the GV cytoplasm contains factors beneficial for reprogramming of round spermatids and embryonic development of ROSI-derived embryos, and GV nucleolus may also have a potential to improve reprogramming of round spermatids.

## Materials and Methods

### Reagents and Animals

All reagents were purchased from the Sigma-Aldrich Chemical Co. (St. Louis, MO, USA) unless otherwise stated. In all studies, BDF1 (C57BL/6 × DBA/2) mice were used to collect oocytes, spermatozoa and round spermatids. 

### Ethics Statements of Animal Care

The animal procedures were approved by Shanghai Jiao Tong University, School of Medicine, and this study was carried out in strict accordance with the National Research Council Guide for Care and Use of Laboratory Animals [SYXK (Shanghai 2007–0025)]. All surgery was performed under sodium pentobarbital anesthesia, and all efforts were made to minimize suffering.

### Preparation of mouse GV cytoplasmic extracts and treatment of round spermatids

Female mice were injected with 7.5 IU of pregnant mare serum gonadotrophin and their ovaries were collected 45 hours later. Fully-grown oocytes were collected in HEPES-buffered CZB [25] medium containing 100 μg/ml dibutyryl cyclic AMP. The oocytes were stored in KSOMaa medium (Specialty Media, Phillipsburg, NJ, USA), containing 100 μg/ml dibutyryl cyclic AMP at 37°C in a humidified 5% atmosphere until use. In order to prepare cytoplasmic extracts, 500 GV oocytes were collected and the entire GVs were removed using a micromanipulator [20] (Movie S1), then the zonae pellucidae were removed using acidic Tyrode’s solution. Enucleated, zona-free oocytes were broken in 5 μl droplets of oocyte lysis buffer, which was HEPES-CZB medium containing 1 mM ATP, 10 mM creatine phosphate, 25 μg/ml creatine kinase, 100 μM GTP and protease inhibitors.

To collect spermatozoa and round spermatids, the epididymis and seminiferous tubules of the testes from 10-week-old male mice were obtained, as described previously [4], except that the cells were suspended in Hepes-buffered CZB medium at room temperature. The plasma membrane of round spermatids were removed using a micromanipulator, and the spermatids were then incubated in 5 μl droplets of the GV cytoplasmic lysates for 45 minutes at 37°C with 100 cell nuclei per lysate. Control spermatids were incubated in 20μl of oocyte lysis buffer as above-mentioned for 45 minutes at 37°C. Spermatozoa and treated round spermatids were then used for ICSI and ROSI. 

### Collection of GV oocyte nucleoli

Nucleoli were collected from GV stage mouse oocytes with a micromanipulator equipped with a piezo drive unit (Prime Tech, Ibaraki, Japan) in modified Hepes-CZB medium supplemented with 0.1 mg/ml dibutyryl cyclic AMP and 7.5 µg/ml cytochalasin B, as previously described with minor modifications [26] (Movie S2). The diameter of the pipette for nucleolus collection was approximately 5 μm. After penetrating the zona pellucida, the injection pipette was positioned against the GV membrane and gentle suction was applied to pull the nucleolus into the mouth of the pipette. The pipette was then slowly withdrawn and when its opening was just outside the vitelline membrane, additional suction was applied to make the nucleolus penetrate the GV membrane and leave the entire contents of the GV within the nuclear envelope.

### Round spermatid injection and oocyte activation

Recipient MII oocytes were collected from superovulated female mice treated with 7.5 IU pregnant mare serum gonadotrophin and 7.5 IU human chorionic gonadotrophin, as previously described [27]. All embryos fertilized by ROSI were subjected to activation with 10 mM SrCl_2_ for 5-6 hours [13,28]. ROSI oocytes were cultured in KSOMaa medium at 37°C in a 5% CO_2_ atmosphere.

### Intracytoplasmic sperm injection

One-microliter aliquots of the suspension were placed in droplets of 10% polyvinyl pyrolidone (PVP)-HEPES-buffered CZB solution in a micromanipulation chamber. ICSI was performed as described [29,30]. Briefly, the sperm head was separated from the tail by applying several piezo pulses to the neck region and the head was then injected into an oocyte. After 10 minutes of recovery at room temperature, the oocytes were cultured in KSOMaa medium at 37°C in a 5% CO_2_ atmosphere. 

### Experimental design

**Figure 1:**
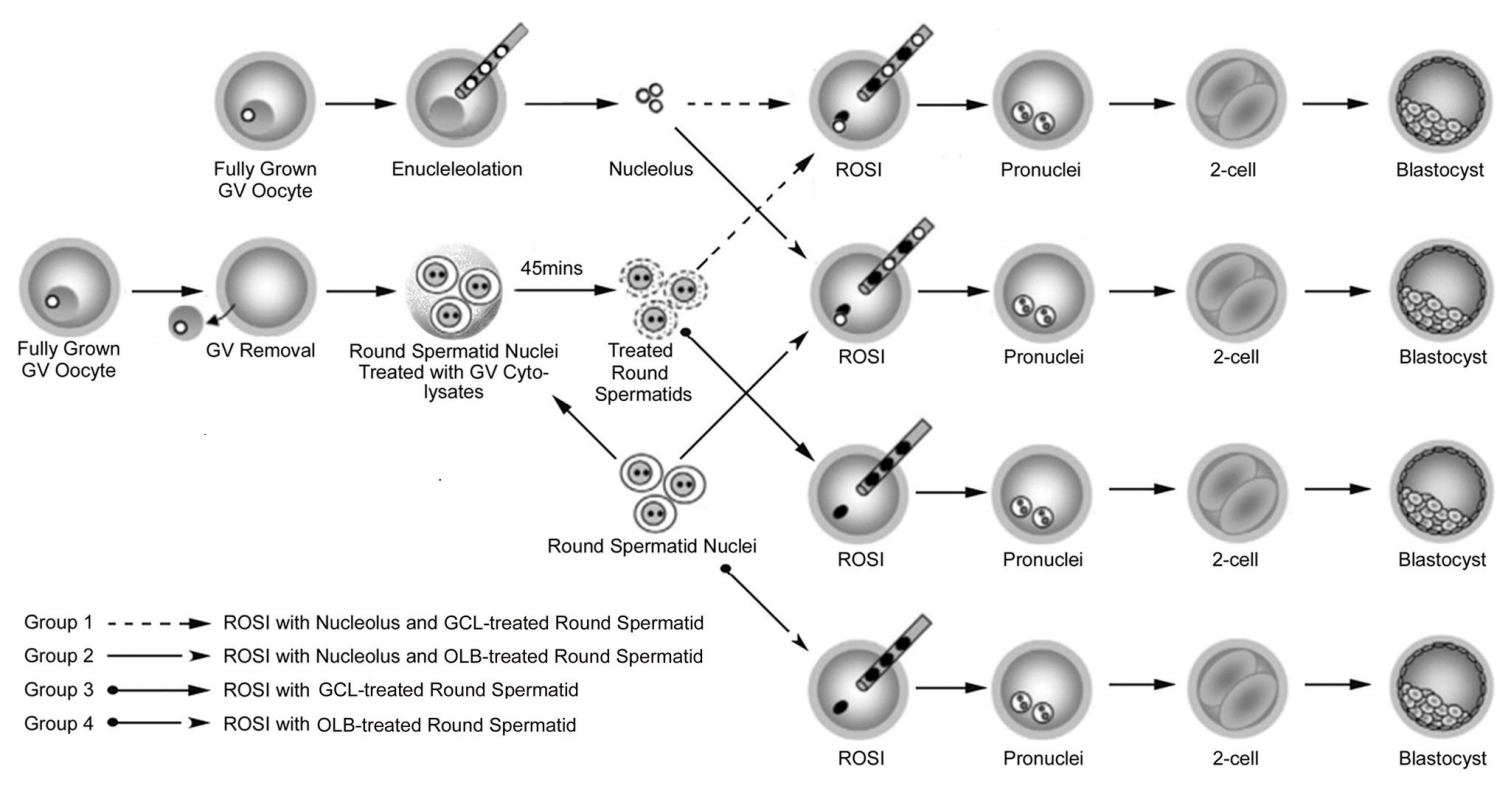
Overview of the experimental design. This study consisted of four experimental groups and one ICSI control group. In Group 1, a round spermatid treated with GV cytolysates and a nucleolus were both injected into an MII oocyte; in Group 2, a oocyte lysis buffer treated-round spermatid and a nucleolus were injected into an MII oocyte; in Group 3, only a round spermatid treated with GV cytolysates was injected into an MII oocyte; and in Group 4, only oocyte lysis buffer treated-round spermatids were injected into an MII oocyte. GCL; GV cytolysate. OLB; Oocyte lysis buffer.

A schematic overview of our experimental design is shown in Figure 1. Our studies included four experimental groups and one ICSI control group. In Group 1, a GV cytolysate treated-round spermatid and a nucleolus were both injected into an MII oocyte; in Group 2, a lysis buffer treated-round spermatid and a nucleolus were injected into an MII oocyte; in Group 3, only a GV cytolysate treated-round spermatid was injected into a MII oocyte; and in Group 4, a lysis buffer treated-round spermatid was injected into an MII oocyte.

### Immunofluorescent analysis of Oct4 protein expression and category of blastocyst quality

ROSI and ICSI blastocysts were fixed and treated as previously described [31]. In brief, the blastocysts were fixed with 4% (w/v) paraformaldehyde in PBS for 40 min at room temperature. Fixed samples were stored in PBS containing 0.3% (w/v) BSA for up to 1 week at 4°C prior to further analysis. For immunofluorescence, samples were permeabilized in PBS containing 0.1% (w/v) Triton X-100 and 0.3% BSA for 30 to 40 min at 37°C. After washing twice with PBS containing 0.01% Triton X-100, they were incubated in blocking solution (PBS containing 150 mM glycine and 0.3% BSA) for 30 min at 37°C. Following blocking, samples were subsequently incubated with the primary antibody rabbit anti-Oct4 (H-134, 1:50, Santa Cruz Biotechnology, Santa Cruz, CA ) and then a FITC- labeled chicken anti-rabbit antibody (1:100; Invitrogen, Grand Island, NY, USA), diluted in blocking solution for 40 min each at 37°C, or overnight at 4°C. Following three 5 min washes, chromatin was stained with 10 µg/ml DAPI (4',6-diamidino-2-phenylindole). Finally, the samples were mounted on slides with anti-fade mounting medium, then examined with a laser-scanning confocal microscope (Zeiss LSM 510 Meta; Carl Zeiss AG, Oberkochen, Germany). Images shown in the result section are representative of at least 40 samples from more than three experimental replicates.

According to the number of *Oct4*-positive blastomere, all the blastocysts were categorized in to Grade A and B groups. Grade A blastocyst has more than 15 *Oct4*-positive blastomeres; Grade B blastocyst has less than 15 but more than eight *Oct4*-positive blastomeres. The remaining blastocyst was without any or has less than eight *Oct4*-positive blastomeres.

### Statistical analysis

The rates of surviving oocytes, embryonic development, pups born, and the percentages of blastocysts with *Oct4*-positive cells at different grades were analyzed using the chi-square test. The data of blastomere were analyzed using student *t* test. Significant differences were accepted when *P* values were less than 0.05.

## Results

### The effect of GV cytolysate treatment and nucleolus injection on the number of blastomere and the expression of Oct4 in ROSI embryos at the expanded blastocyst stage

**Figure 2:**
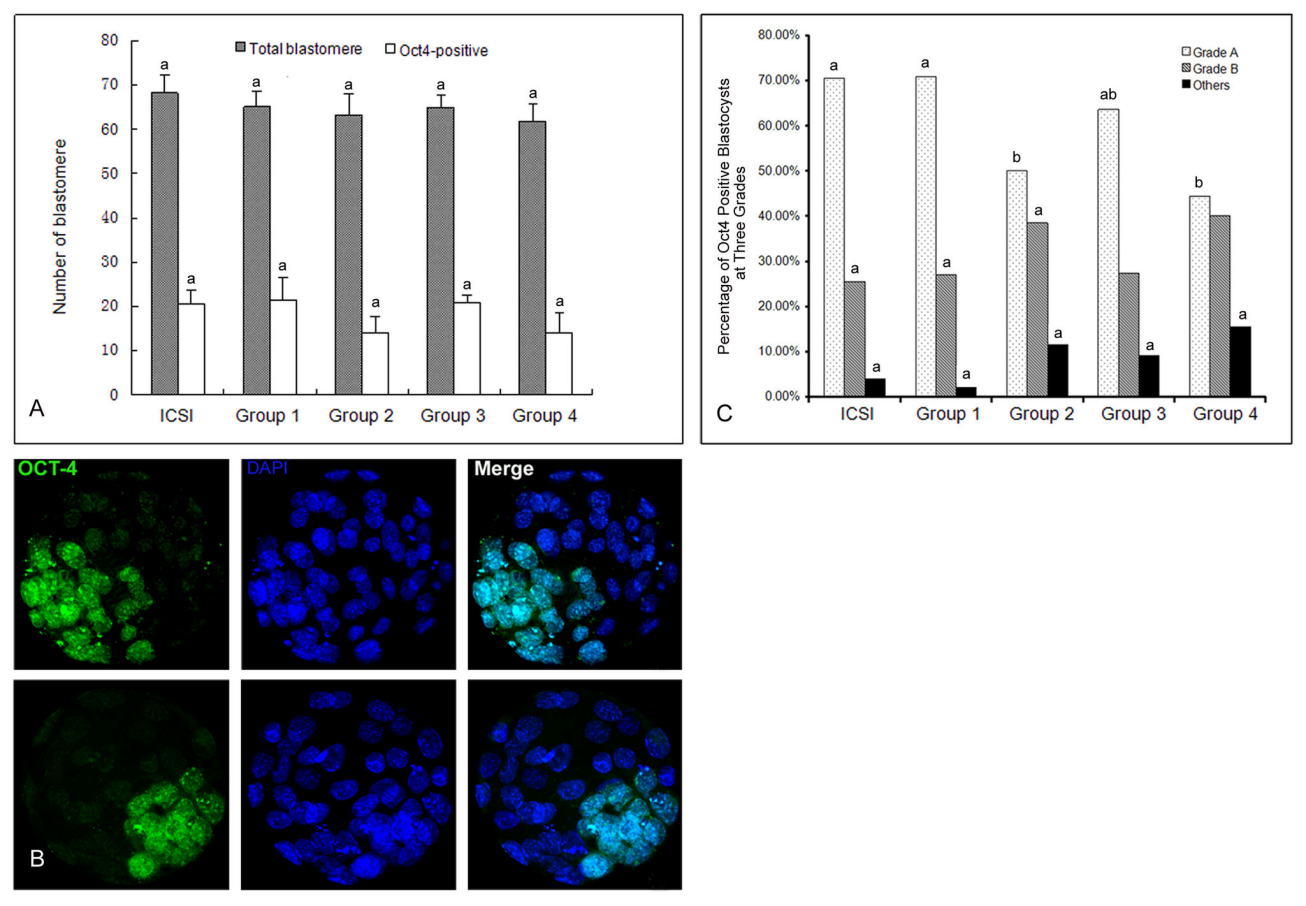
*Oct4* expression in expanded blastocysts and the percentage of blastocysts expressing *Oct4*. The total blastomeres and the blastomeres expressing *Oct4* in expanded blastocysts from ICSI control and four ROSI groups; Bars represent means ± SD (A). Representative images of *Oct4* expression in expanded mouse blastocysts derived from ROSI or ICSI (B); The upper panels show an example of a Grade A blastocyst with more than 15 Oct4-positive blastomeres, and the lower panels show an example of Grade B blastocysts with between eight and 15 Oct4-positive blastomeres. Oct4 expression is shown in green and DAPI-stained nuclei are shown in blue; Scale bar: 20 μm. The proportion of Grade A, Grade B and other blastocysts (less than 8 Oct4-positive blastomeres) are also shown for each experimental group and the ICSI control group (C). The values on the same color columns bearing different letter on top were significantly different at *P* < 0.05.

*Oct4* is a specific gene marker for the inner cell mass (ICM) at the expanded blastocyst stage. To assess the quality of blastocysts produced by ROSI, the ICM and total cell numbers were evaluated to determine *Oct4* expression. There was no significant difference in total blastomere and *Oct4* positive blastomere at the blastocyst stage between the four groups (*P*>0.05; Figure 2A and Table S1).

To examine the relationship of GV cytolysate treatment and (or) nucleolus injection with the expression of *Oct4* in expanded blastocysts, we categorized the expanded blastocysts into three grades based on the numbers of *Oct4*- positive ICM cells (Figure 2B). Our results showed that the percentage of blastocysts containing ICM with *Oct4*-positive blastomeres (more than 15 *Oct4*-positive blastomeres) was significantly different in the ICSI and four experimental ROSI groups (Figure 2C and Table S1). High-quality (grade A) blastocysts were detected at a rate of 70.59%, 63.63% and 70.3% in the ICSI, and GV cytolysate treatment Group 3 and Group 1 blastocysts, respectively. Grade B blastocysts were detected at a rate of 25.49%, 27.27% and 27.08% in the ICSI group, Group 3 and Group 1, respectively. Grade A embryos were detected at a rate of 50% and 44.44% in the groups without GV cytolysate treatment (Group 2 and 4, respectively). The occurrence of grade B embryos in these two groups was 38.46% (Group 2) and 40.00% (Group 4).

### The effect of GV cytolysate treatment and nucleolus injection on ROSI efficiency

The rates of pronuclear formation and embryo development to the two-cell stage were not significantly different within the ICSI control group and four experimental groups, which were 95.23%, 85.83%, 84.54%, 88.39%, 90.08% and 93.00%, 86.41%, 92.68%, 87.88%, 83.05%, respectively (*P* >0.05; Table 1). Moreover, the rates of embryo developed to the four-cell stage were also not significantly different within the four experimental groups, which were 84.46%, 90.24%, 84.84% and 81.35%, respectively (*P* >0.05; Table 1). 

**Table 1:** Effect of GV cytolysate and nucleolus on the development and birthrate of ROSI-derived embryos.

Groups	No. of oocytes	Replicates	No. of embryos developed to (%)					No. of full-term offspring (%)
			Zygote	2-cell	4-cell	Morula/blastocyst		
ICSI	105	3	100(95.23)^a^	93(93.00)^a^	ND	72(77.42)^a^	41(56.94)^a^	
Group 1	120	4	103(85.83)^a^	89(86.41)^a^	87(84.46)^a^	69(77.53)^a^	38(55.07)^a^	
Group 2	97	3	82(84.54)^a^	76(92.68)^a^	74(90.24)^a^	42(55.26)^b^	13(30.95)^b^	
Group 3	112	3	99(88.39)^a^	87(87.88)^a^	84(84.84)^a^	61(70.11)^a^	31(50.82)^a^	
Group 4	131	4	118(90.08)^a^	98(83.05)^a^	96(81.35)^a^	49(50.00)^b^	14(36.73)^b^	

Difference of the superscripts within columns represents the significant difference at *P* < 0.05 (chi-square test). ND; No detection.

While all four experimental groups yielded live offspring, oocytes which were co-injected with GV cytolysate treated-round spermatids and nucleoli or injected with GV cytolysate treated-round spermatids alone yielded more live offspring than those were obtained from the lysis buffer treated-round spermatids (Table 1). Furthermore, there was no difference in the proportion of offspring produced in Group 1 (55.07%) and Group 3 (50.82%) versus the ICSI group (56.94%; *P* > 0.05). Moreover, the developmental rates of blastocysts derived from the cytolysate treated-round spermatid groups (Group 1 [77.53%] and Group 3 [70.11%]) were comparable with the ICSI control group (77.42%; *P* > 0.05), and were significantly higher than that of lysis buffer treated-round spermatid (*P* < 0.05; Group 2 [55.26%] and Group 4 [50%]).

## Discussion

The extracts of *Xenopus* oocytes can remodel mammalian somatic cell genomes [32-36], although GV and MII stage oocyte cytoplasm from amphibians and mammals have shown different reprogramming capabilities [20,37,38]. In their mouse cloning study, Bui et al. found that the GV stage cytoplasm had the ability to remodel somatic cell nuclei through altering its chromatin structure and epigenetic modifications, suggesting that genomic reprogramming factors in the cytoplasm of GV stage oocytes have the ability to improve the efficiency of producing cloned mice offspring [20]. More recently, it was demonstrated that GV cytolysates could provide the necessary regulatory components required for inducing somatic cell nuclear reprogramming and for altering the differentiation status of non-embryonic cells [19]. 

In the present study, we used GV cytolysates to reprogram round spermatid nuclei in mice, and found that round spermatid nuclei could be manipulated epigenetically using mammalian GV cytolysates as a method to enhance round spermatid reprogramming. Our results demonstrate that embryos derived by ROSI following treatment with GV cytolysates had a higher proportion of pluripotent cells in the ICM (as evidenced by Oct4 expression), and increased blastocyst development rate. Thus, ROSI-derived embryos from round spermatid nuclei treated with GV cytolysates resulted in high-quality blastocysts. 

The second factor influencing embryonic development examined in this study was the nucleolus. The nucleolus is an important organelle whose main functions are ribosome biogenesis and transcription of ribosomal RNA genes [39]. Indeed ribosomal RNA gene activation and the associated nucleolus formation may be used as a marker for the activation of the embryonic genome in mammalian embryos, and thus can be used to evaluate the developmental potential of embryos originating from varied assisted reproduction protocols [40,41]. Nucleoli, also called the nucleolus precursor body-NPB, play an important structural and chromatin organizing role, especially for the pericentric heterochromatin and centromeres that associate with this nuclear body [42]. Recent studies have demonstrated the role of epigenetic mechanisms in establishing pericentric heterochromatin and centromere function, serving to emphasize the potential of nucleolus transplantation therapy for assisted reproduction [24,42].

Regarding the male gamete, the nucleolus in the spermatozoon is eliminated during spermiogenesis, thus the oocyte nucleolar material is essential for the reassembly of newly formed nucleoli in both female and male pronuclei [21-23].

During the process of somatic cell nuclear transfer, the ooplasm of oocytes after GV breakdown and the ooplasm of mitotic zygotes support development after nuclear transfer, whereas the ooplasm of oocytes before GV breakdown and ooplasm of interphase zygotes do not [16,43]. When the pronuclear membrane and chromatin of interphase zygotes are selectively removed but the nucleolus is left in the cytoplasm, ooplasm supports development of somatic cell nuclear transfer embryos [44]. These findings suggest that an undefined activity or factor(s) in the GV or pronuclei of zygotes facilitates reprogramming, and that the nucleolus is essential for reprogramming [43,44]. 

Finally, we obtained live offspring after embryo transfer from all four experimental groups, with higher birthrates in GV cytolysate treatment groups. However, the development of ROSI-derived embryos from oocytes which were co-injected with GV cytolysate treated-round spermatids and nucleoli was higher than that of oocytes which were injected with GV cytolysate treated-round spermatids alone, but there was no significant difference between the birthrates, which directly confirms that the reprogramming capability to round spermatids was derived from GV cytolysates, but not GV nucleolus.

In conclusion, our data suggests that factors present in the GV cytoplasm, not in the nucleus, can improve the reprogramming of round spermatid nuclei. We also speculate that the extra nucleolus we injected might have a beneficial effect on the development of ROSI-derived embryos, but did not significantly improve the reprogramming of round spermatids, because we cannot exclude a possible contribution of the nucleoli to cell reprogramming. Thus, some reprogramming factors are evidently present in GV oocyte cytoplasm and could significantly facilitate ROSI technology, while the nucleolus in GV seems also having a potential to improve reprogramming of round spermatids.

## Supporting Information

Movie S1 **Removal of GV from fully-grown oocyte.** After the fully-grown GV oocytes were collected, in order to prepare cytoplasmic extracts, the entire GVs were removed using a micromanipulator in modified Hepes-CZB medium supplemented with 0.1 mg/ml dibutyryl cyclic AMP and 7.5 µg/ml cytochalasin B. The inner diameter of the pipette for nucleolus collection was approximately 5 μm.
(MP4)

Movie S2 **Collection of GV oocyte nucleoli.** Nucleoli were collected from GV stage mouse oocytes with a micromanipulator equipped with a piezo drive unit (Prime Tech) in Hepes-CZB medium supplemented with 0.1 mg/ml dibutyryl cyclic AMP and 7.5 µg/ml cytochalasin B by using a pipette with 10~12 μm inner diameter.
(MP4)

Table S1 **Oct4 positive blasomeres in expanded blastocysts and the percentages of blastocysts expressing Oct4.** Numbers of blastomere and percentages of blastocysts were analyzed using student *t*- and chi-square test, respectively. Values with different superscripts within columns are significantly different at *P* < 0.05. SD; Standard deviation.
(DOC)
