# Supplementary material for: Reprogramming of Round Spermatids by the Germinal Vesicle Cytoplasm in Mice
Source: PLoS One. 2013 Oct 22;8(10):e78437. doi: 10.1371/journal.pone.0078437 (PMC3805568; doi:10.1371/journal.pone.0078437)
Supplement: Table S1 — Oct4 positive blasomeres in expanded blastocysts and the percentages of blastocysts expressing Oct4. Numbers of blastomere and percentages of blastocysts were analyzed using student t- and chi-square test, respectively. Values with different superscripts within columns are significantly different at P < 0.05. SD; Standard deviation. (DOC) [file pone.0078437.s003.doc]

**Table S1. *Oct4* positive blasomeres in expanded blastocysts and the percentages of blastocysts expressing *Oct4***

| Groups | No. of blastocysts | Total blastomere (Means ± SD) | No. of *Oct4* positive blastomere (Means ± SD) | No. of blastocysts (%) at | | |
| --- | --- | --- | --- | --- | --- | --- |
|  |  |  |  | Grade A | Grade B | Others |
| ICSI  Group 1  Group 2  Group 3  Group 4 | 51  48  52  44  45 | 68.20±4.21^a^  65.08±3.52^a^  63.21±4.91^a^  64.88±3.01^a^  61.87±3.76^a^ | 20.58±3.15^a^  21.32±5.12^a^  14.09±3.55^a^  20.96±1.56^a^  14.12±4.35^a^ | 36(70.59)^a^  34(70.83)^a^  26(50.00)^b^  28(63.63)^ab^  20(44.44)^b^ | 13(25.49)^a^  13(27.08)^a^  20(38.46)^a^  12(27.27)^a^  18(40.00)^a^ | 2(3.92)^a^  1(2.08)^a^  6(11.54)^a^  4(9.09)^a^  7(15.56)^a^ |

### Numbers of blastomere and percentages of blastocysts were analyzed using student *t*- and *chi*-square test, respectively. Values with different superscripts within columns are significantly different at *P* < 0.05. SD; [Standard deviation](http://www.google.com.hk/url?sa=t&rct=j&q=SD，全称+means&source=web&cd=3&cad=rja&ved=0CDcQFjAC&url=http%3A%2F%2Fen.wikipedia.org%2Fwiki%2FStandard_deviation&ei=545DUu-HIcbpiAeaxYGoDA&usg=AFQjCNFtYyZ796mAmi78GBXFEt41u34oow).
